# The Numerical Fatigue Life Analysis of a Conformal HPDC Mould Core Additively Manufactured from Maraging Steel

**DOI:** 10.3390/ma16010365

**Published:** 2022-12-30

**Authors:** Jarosław Piekło, Aldona Garbacz-Klempka, Andriy Burbelko

**Affiliations:** Faculty of Foundry Engineering, AGH University of Science and Technology, Mickiewicza 30, 30-059 Krakow, Poland

**Keywords:** die casting, thermal fatigue, conformal cooling, conventional cooling, temperature analysis, strength analysis, microstructural analysis

## Abstract

This paper presents the results of a stress analysis and fatigue life calculation of an HPDC mould core. The calculations were performed using Abaqus and fe-safe software. The numerical model of a core cooled by a conformal channel was based on an existing and working counterpart made of additively manufactured high-strength 1.2709 maraging steel. This study shows that the conformal channel results in a lower average core temperature as compared to the temperature of the same core shape cooled by the conventional method. The course of the stress changes during the mould cycle was also determined. It was found that stresses on the core surface caused the cyclic compression and tension of the material. The necessary strength tests of 1.2709 steel produced by selective laser melting (SLM) within a temperature range of 25 to 550 °C, which were necessary to define the fatigue coefficients by the Seeger approximation method, were also carried out in this study, along with metallographic tests of the fractures of the specimens. Based on the multiaxial fatigue criterion and using the maximum principal deformation hypothesis, the fatigue life of the core and channel surfaces was determined. Based on the calculations, it was shown that crack initiation on the channel surface can occur earlier than on the outer surface of the core.

## 1. Introduction

High Pressure Die Casting (HPDC) moulds are most often designed for the high-volume production of small and medium-sized thin-walled aluminium alloy castings. The efficiency of the process depends on the cycle time of the pressure machine and the number of cavities in the mould, and its profitability increases as the number of castings produced increases. A properly designed mould-cooling system has a major impact on improving casting properties. Eliminating or at least reducing the hot spots in the casting reduces the level of shrinkage porosity, and an increase in the cooling rate leads to grain refinement [1]. Optimisation of the cooling system is possible by replacing the conventional system of rectilinear channels with a system of conformal channels whose trajectories and cross-sections are matched to the shape of the cooled surface [2,3,4,5,6,7,8]. Pressure mould parts with a conformal system can only be manufactured using additive methods (for example, SLM) [9].

The following phases can be distinguished during HPDC cycles: injecting the alloy after the mould has been closed, pressing the liquid phase under high pressure to compensate for any shrinkage of the alloy during crystallisation, opening the mould and extracting the resulting bundle (castings with a gating system) from the mould, applying a protective coating on the moulding surfaces of the pressure mould by spraying with a water suspension, and drying. The temperature of the forming elements of the pressure mould varies over a wide range during the working cycle. These changes occur on the moulding surfaces that are in direct contact with the casting alloy.

In his study, Norwood [10] showed that the surface of the pressure mould insert heated up to 400–450 °C in about 1 s and cooled down to 150–200 °C in about 20 s when the initial temperature of the injected Al-Si8Cu3Fe alloy was 750 °C. On the other hand, Piekło [11] experimentally determined that, for an initial alloy temperature of 680 °C at a distance of 1 mm from the moulding surface for a cycle that lasts 30 s, the mould temperature oscillated between 230–390 °C. Due to the limited thermal conductivity of the mould material, those areas that are further away from the forming surfaces do not heat up to this level, and the amplitude of the temperature oscillation is smaller. According to Klein et al. [12], the temperature of the mould cavity surface reaches about 500 °C, while the area located 30 mm from the cavity surface heats up to only slightly above 200 °C. Ružbarský et al. [13], on the other hand, pointed out that the temperature of the moulding cores is higher than that of the moulding insert material; this was related to the smaller ratio of the core mass to the area of its working surface. The data cited above refer to the steady-state operation of the equipment, and the differences were due to variations in the initial melt temperature, cycle time, casting wall thickness, and different mould-cooling systems.

The results of the authors’ numerical calculations in [14] showed large differences in the temperature and stresses in the mould during the initial few cycles of the operation and the subsequent cycles when the mould temperature was already stabilised.

The changes in the mould’s surface temperature that occur during the operating cycles and the temperature gradient inside the mould generate a time-varying thermal stress field. This phenomenon is particularly intense in the area close to the mould cavity surface. The stresses on the surface of the mould cavity change not only their values, but also their directions of action during the cycle, causing the phenomenon of the thermal fatigue of the mould material. An analysis of the biaxial stress state occurring on the mould cavity surface showed that the components of the stress state during the melt injection and cooling induced the phenomenon of compressing the mould material that is in close vicinity of the mould surface, while the stresses changed their signs to positive during the spraying. This means that tensile forces act on the surface layer of the mould material [15,16,17]. The total deformation of the mould material under different temperature changes derives from the thermal deformation and mechanical deformation (which is due to the plastic and elastic strain). If the component to be heated cannot extend freely, the entire thermal deformation is assumed to be transformed into mechanical strain [18]. The above case largely corresponds to the boundary conditions of the attachment of the individual parts of the pressure mould. Periodic changes in temperature and stress occurring particularly intensively at the mould surface induce the phenomenon of fatigue—the consequence of which is the initiation of cracks.

The results of a study by A. Klobcar [19] showed significant differences in the thermal fatigue resistance of different steels used for pressure mould parts. The heat-treated H13 steel specimens that were tested had a small number of very long cracks, although there was no significant decrease in the hardness of the material. Samples from H11 steel had slightly longer cracks and a greater decrease in hardness than the H13 steel. In contrast, the maraging steel samples had a high density of short cracks, not very long cracks, and a significant decrease in hardness. The study also found that high-yield-strength materials showed increased crack density, while low-yield-strength materials were characterised by longer cracks. The above fact is significant due to the very high yield strength of the maraging steel obtained by SLM [20]. The increase in the crack propagation velocity is also favoured by surface phenomena occurring at the ‘liquid metal-mould’ interface—especially the oxidation of the surfaces of the existing cracks and their filling by the liquid casting alloy during the injection [21]. The same authors also highlighted the effect of the softening of H13 steel during successive thermal fatigue cycles on the increase in the crack propagation speed, and Long et al. [22] were able to compare its damage rate with that predicted on the basis of numerical calculations thanks to a replaceable pressure-moulded insert.

Observations and analyses of crack formation for single-unit design cases that are carried out directly during an operation on material taken from a pressure mould are of great value, as a fatigue-life assessment of parts is among the main challenges facing the widespread use of SLM-manufactured parts [23]. Yadollahi et al. [23] argued that the damage evolution of SLM materials under cyclic loading conditions was directly influenced by contaminants from the SLM process itself. Among the many different causes of damage evolution under cyclic loading, voids are the main life-limiting factor, and the dominant fatigue-cracking mechanism in additively manufactured metals is the propagation of initiating cracks on their surfaces. The random locations, shapes, and sizes of voids are the main reason for the large scatter in fatigue data. In contrast, Dastgerdi et al. [24] argued that surface defects were more detrimental to fatigue life than internal defects. The interaction effect of internal defects and surface roughness has a very strong influence on the fatigue life of SLM specimens; hence, the distance of voids from the part surface and their distribution should be investigated. Also, the orientation of an SLM part structure relative to a work platform surface can generate an anisotropic structural response—especially in terms of fatigue behaviour [25]. Fatigue life is also affected by the surface roughness of an SLM-made part. Yadollahi et al. [26] and Pegues et al. [27] showed that knowledge of the statistical distribution of the defect size and shape provided the ability to predict the fatigue life spread of SLM materials. Furthermore, they found that the maximum depth of the profile defect of an unmachined surface could be used as a suitable parameter to predict the fatigue life of SLM materials. The classical description of crack formation assumes that there are three stages of crack development: crack initiation, stable propagation, and the failure phase. The first of these belongs to fatigue issues, while the second and third stages can be predicted using fracture mechanics equations. The criterion for distinguishing the applicability of fatigue and fracture mechanics theories is the crack length (which is 0.5 to 1.0 mm). There is a biaxial state of stress on the surface of the mould cavity; hence, the biaxial fatigue criterion should be used to describe this phase of the crack development [28].

This paper focuses only on the first stage of crack development, assuming that crack development during the propagation phase eliminates a mould from use. The purpose of the research that has been undertaken in this study was to determine the state of the stress in a mould core made by the SLM method and cooled by a conformal channel. A channel made by the SLM method in a mould core differs significantly from the classical solution of a drilled linear channel. In addition to the shape, the differences relate to the dimensions of the channel cross-section and the distance from the contact surface of the core to the casting alloy. In the case of a conformal channel, this distance is much smaller than for a linear channel. The conformal channel lowers the average core temperature; however, it can cause a local increase in stress values by increasing the cooling intensity. The paper also attempts to determine the number of mould-filling cycles until cracks are initiated in the core based on the determined stress field by using a multiaxial fatigue criteria and maximum principal strain analysis. Metallographic and strength tests were performed to determine the temperature above which the SLM-printed 1.2709 steel loses its strength properties. The results of these tests were used to define the material properties that were assumed in the numerical model of the core.

## 2. Materials and Methods

The test specimens were made by the SLM method from maraging steel powder with the trade symbol “MS1” obtained by gas atomization (recommended by EOS GmbH). The chemical composition of the powder corresponded to the steel that was designated 1.2709, DIN X3NiCoMoTi18-9-5, which is a high-strength martensitic steel for use at elevated temperatures and strengthened by aging. The chemical composition of the powder determined during the testing is shown in Table 1.

**Figure 1:**
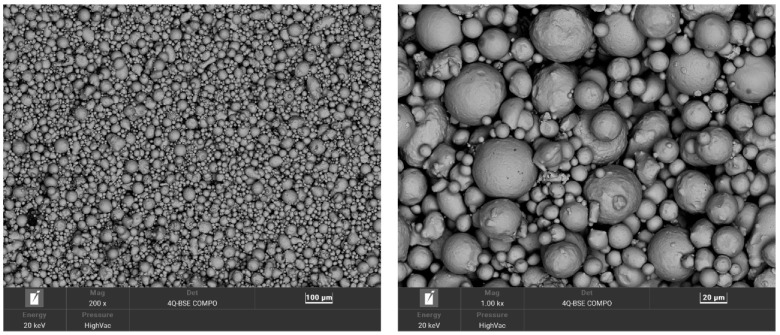
The powder morphology of 1.2709 steel shown at two different image magnifications.

The chemical composition of the powder was determined by spectral analysis using an energy-dispersive X-ray fluorescence spectrometer (ED-XRF) SPECTRO MIDEX. The powder used in the study was a powder that had already been previously used for the SLM printing of the parts. Figure 1 shows the morphology of the powder used.

The samples were made using the SLM method in the chamber of an EOS M290 device in an argon atmosphere. The following printing parameters were used: layer thickness—40 µm; laser power—380 W; laser beam diameter—80 µm; laser speed—1500 mm/s; hatch space—90 µm. The axes of the printed samples were located perpendicularly to the surface of the base plate. The heat treatment of the samples consisted of ageing for six hours at 490 °C. Heating and cooling of the samples was carried out at a rate of 100 K/h. The samples for testing at ambient temperature after SLM fabrication were not machined. The measuring part of the samples was 30 mm long and 5 mm in diameter.

An MTS extensometer with a 20 mm base was used to measure the strain. The static tensile test was carried out on an MTS 810 tensile machine. Static tensile tests were carried out at 200, 300, 470, 500, and 550 °C on a WTW-300E III tensile machine, which was equipped with a GW-1200A split-chamber temperature furnace and a YYU-10/50-SH extensometer. The temperature measurements in the furnace were made by three thermocouples that were placed in the furnace chamber. The countdown of the annealing time of the samples at the set temperature began when the difference between the individual measurement results did not exceed 1 K. After 12 min of annealing, the extensometers’ readings were reset before the measurements began.

Observations of the fracture surfaces of the strength specimens were carried out by scanning electron microscopy (SEM) using a high-resolution Tescan Mira microscope with an FEG electron source. The topography of the sample was examined using solid-state detectors. The beam energy was 20 keV. The surface was observed under backscattered electron contrast (BSE and BSE COMPO). The studies were conducted at magnifications of 50 and 2000×.

## 3. The Thermal-Stress-Fatigue Model of Phenomenon

The HPDC process repeats cyclically. In each cycle, there are several characteristic phases such as the mould closure, the alloy filling, the solidification and cooling of the alloy, the opening of the mould and removal of the casting, the spraying of the release liquid, and another mould closure. During the working cycle of making a casting, the surface of the mould cavity is alternately in contact with the casting alloy (in the liquid and solid states), air, sprayed water suspension, and forced-air flow during drying. The cyclic temperature changes stabilise only after several (or even a dozen) castings have been made. In the present study, an analysis of the core life was carried out using three numerical models: thermal, stress, and fatigue. Numerical models and calculations were performed using Abaqus and fe-safe programs. The complexity of the course and, thus, the description of the HPDC process required the introduction of a number of assumptions to simplify the calculations (described below).

### 3.1. The Thermal Model

In the thermal model, the duration of one HPDC cycle was assumed to be 35 s. Table 2 shows the division of the cycle into the stages that were considered in the numerical model and gives their durations.

The duration of the individual stages that were assumed in the model was the same as during the production cycles of castings of the same shape made in a two-cavity mould with conformal cores of 1.2709 steel powder made using the SLM method. Only one mould cavity was included in the analysed model. The geometrical models consisted of a core, casting or mould cavity (depending on the stage number), and a half-space model representing the other parts of the mould that were relevant for heat transfer in the vicinity of the casting and core. A ‘surface to surface contact’ model was used to describe the thermal boundary conditions of the heat transfer between the mould and the casting surfaces—the mathematical form of which is defined by Equation (1):

(1) $$q=h_{\mathrm{int}}\cdot \left(T_{Surf1}-T_{Surf2}\right),$$

where *q* (W/m^2^) is the heat flux across the interface, h_int_ (W/m^2^K) is the interfacial heat transfer coefficient (IHTC), and T*_Surf_*_1_ (°C) and T*_Surf_*_2_ (°C) are the temperatures of the two surfaces (sides) of the interface.

In turn, the cooling of the mould surfaces at different stages of the cycle can be described by Equation (2). This includes cooling with liquid that flowed in the cooling channels throughout the cycle, with atmospheric air after the casting is removed and before the mould is closed (Steps 2 and 4), and during the lubricant spraying (Step 3):

(2) $$q=h_{Surf}\cdot \left(T_{Surf}-T_{\infty }\right),$$

where *q* (W/m^2^) is the heat flux, h*_Surf_* (W/m^2^K) is the surface film coefficient IHTC, and T*_Surf_* (°C) and T_∞_ (°C) are the surface and environmental temperatures, respectively. Table 3 gives the values of the h_int_ and h*_Surf_* coefficients used in Equations (1) and (2) for Cycle Stages 1 through 4.

In the heat transport model, the temperature-dependent properties of the AlSi9Cu3 alloy were assumed. Table 4 summarises the values of the thermal conductivity, density, and specific heat used in the calculations. For the SLM 1.2709 steel, it was assumed that these values were invariant over the temperature range occurring in the mould. The calculations assumed an initial alloy temperature of T_init_1_ = 680 °C and an initial core temperature of T_init_2_ = 50 °C. It was assumed that the cycling of the core temperature stabilised after five cycles. After five cycles, the core temperature ranged from approximately 250–300 °C. The core temperature calculated after the fifth cycle was taken as the initial temperature in the analysis of the stress state and fatigue life of the core.

**Figure 2:**
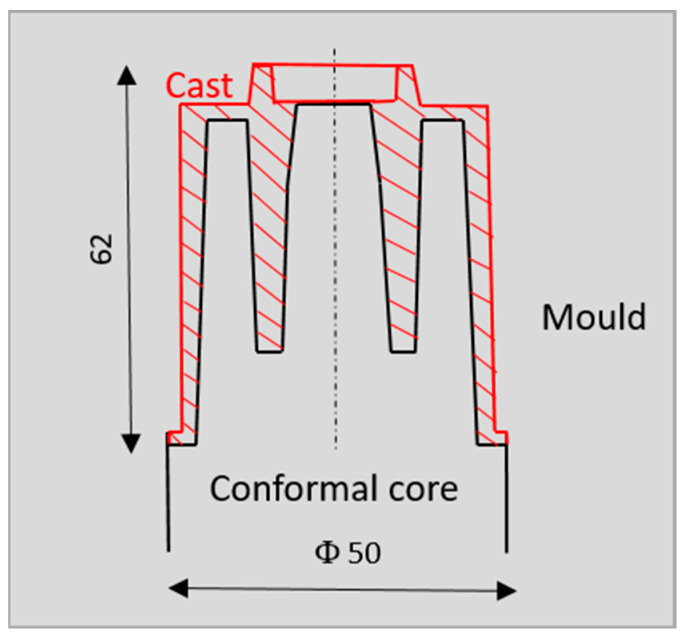
The position of the mould core, mould, and casting in the geometrical model.

The relative positions of the mould core and the casting in the simplified geometric model are shown in Figure 2.

### 3.2. The Stress Model

The stress model took the geometry of the casting and the conformal core into account. Its attachment was determined by taking away degrees of freedom on the mould’s contact surfaces. There was a mechanical contact between the surface of the casting and the surface of the core. The elastic modulus E of the AlSi9Cu3 alloy above a liquidus temperature of 580 °C was assumed to be 100 MPa, while below a solidus temperature of 570 °C, it was 68,000 MPa. The thermal stresses in the core were calculated at 0.5 s intervals based on the pre-determined temperature fields imported from the thermal model. The core model assumed an elastic–plastic material model with linear strengthening and a temperature-dependent elastic modulus and yield stress. Table 5 summarises the mechanical material properties adopted in the calculations for the conformal core. The same geometry and number of non-linear tetrahedron elements were used for both the thermal and stress models. For the thermal model, these were ten-node DC3D10 elements, and for the stress model—C3D10 [29].

### 3.3. The Fatigue Model

The issue of the fatigue life of the core was analysed based on calculations carried out using Abaqus and the fe-safe program that was integrated with it. Based on the analysis of the stress field present in the core, the fatigue cycle of the material was determined. The fatigue cycle was determined by the stress field in the core 2.5 s after the metal injection and by the stress field after 23 s. After 2.5 s, the compressive stress on the core surface reached a maximum value, while after 23 s, the tensile stresses had a maximum value as a result of the rapid cooling of the core surface with the sprayed liquid. In the fatigue model, the values of elastic modulus E, yield strength R_p0.2_, and ultimate tensile strength UTS as a function of the temperature (collected in Table 5) were used to describe the material properties.

The conventional strain-life parameter coefficients used in the fatigue-life-prediction equations are collected in Table 6. The values of these coefficients were determined using the Seeger approximation method [30]. The Seeger approximation method uses the results of a static tensile test to determine fatigue coefficients and is applicable for low-carbon steels. The coefficients were calculated from the results of the tensile tests using the following relationships: fatigue-strength coefficient σ*_f_*_′_ = 1.5 UTS, fatigue-ductility coefficient *f*′ = 0.59a and a = 1.375 − 125*UTS/E), strain-hardening coefficient *K*′ = 1.65 UTS. In the Seeger approximation method, the coefficients b and c have constant values; fatigue-strength exponent *b* = −0.087, and fatigue-strain exponent *c* = −0.57. Maximum principal strain analysis was used to determine the number of *N_f_* cycles followed by crack initiation on the core or channel surface. This multiaxial fatigue criterion proposes that fatigue cracks initiate on planes which experience the largest amplitude of principal strain. Stress results from an elastic FEA are required. A multi-axial elastic-plastic correction is used to calculate the stress-strain relationship from the elastic FEA stresses. The fatigue life is calculated on 18 planes, which are spaced at 10 degrees increments on each plane. Cycles of normal strain ∆ε_1_ are extracted and corrected for mean stress σ*_m_*. The fatigue life is the shortest life calculated for the series of planes. The algorithm uses the strain-life curve defined by the following equation:

(3) $$\frac{\Delta \varepsilon_{1}}{2}=\frac{\sigma_{f}^{\prime }}{E}{\left(2N_{f}\right)}^{b}+\varepsilon_{f}^{\prime }{\left(2N_{f}\right)}^{c}$$

In the fatigue model, the core surface roughness values were assumed to be as follows: the roughness of cooling channel surface *R_a_* = 10 μm and the roughness of the outer surface of the core *R_a_* = 0.4 μm.

## 4. Results

### 4.1. Strength Test Results

**Figure 3:**
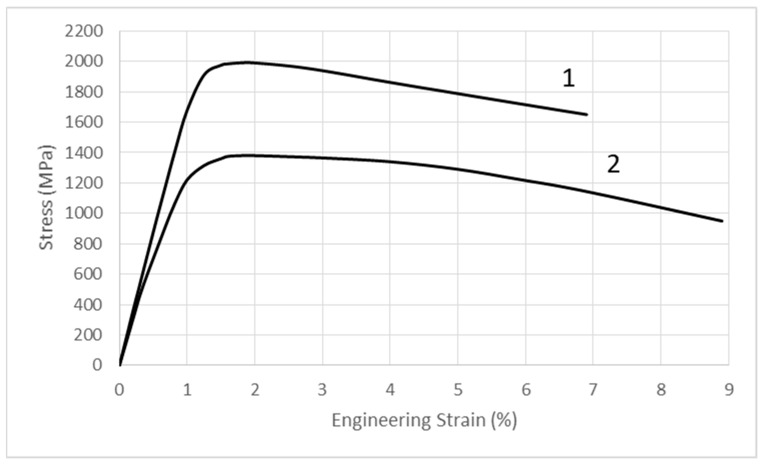
Typical stress-strain curves of the tested steel: 1—25 °C; 2—500 °C.

**Figure 4:**
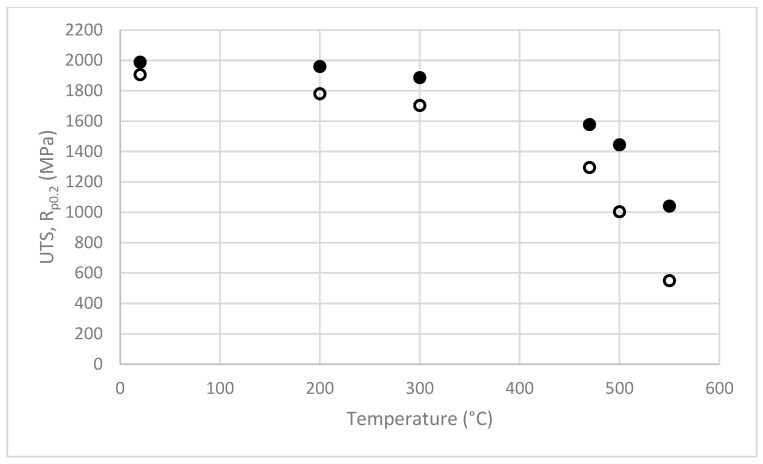
The relationship of UTS (full circles) and R_p0.2_ (empty circles) of tested steel within the temperature range of 20° to 550 °C.

Tensile tests of the SLM-manufactured 1.2709 steel were carried out within a temperature range of 20–550 °C. On the basis of the tests, the properties of the steel (such as elastic modulus E, yield strength R_p0.2_, and tensile strength UTS) were determined. Figure 3 shows the typical stress–strain curves of the tested steel at 25 and 500 °C. The steel had a high yield strength R_p0.2_ that was close to the UTS tensile strength (Figure 4), especially within a temperature range of 25 to about 470 °C. Based on tests, it was shown that, at 25 °C, the value of Rp_0.2_ = 0.96 UTS, at 300 °C—Rp_0.2_ = 0.9 UTS, and at 470 °C—Rp_0.2_ = 0.82 UTS. Above 470 °C, the difference between yield strength Rp0.2 and ultimate tensile strength UTS increased rapidly.

Figure 4 shows the changes in the yield and tensile strengths of the tested steel as a function of the temperature. The large decrease in strength above approximately 470 °C was evident.

### 4.2. The Fracture Surface Morphology

After the tensile tests within a temperature range of 25–550 °C, the fracture surface morphology of the samples was analysed using a scanning microscope.

**Figure 5:**
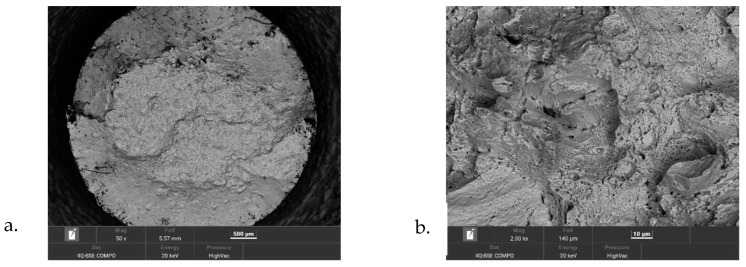

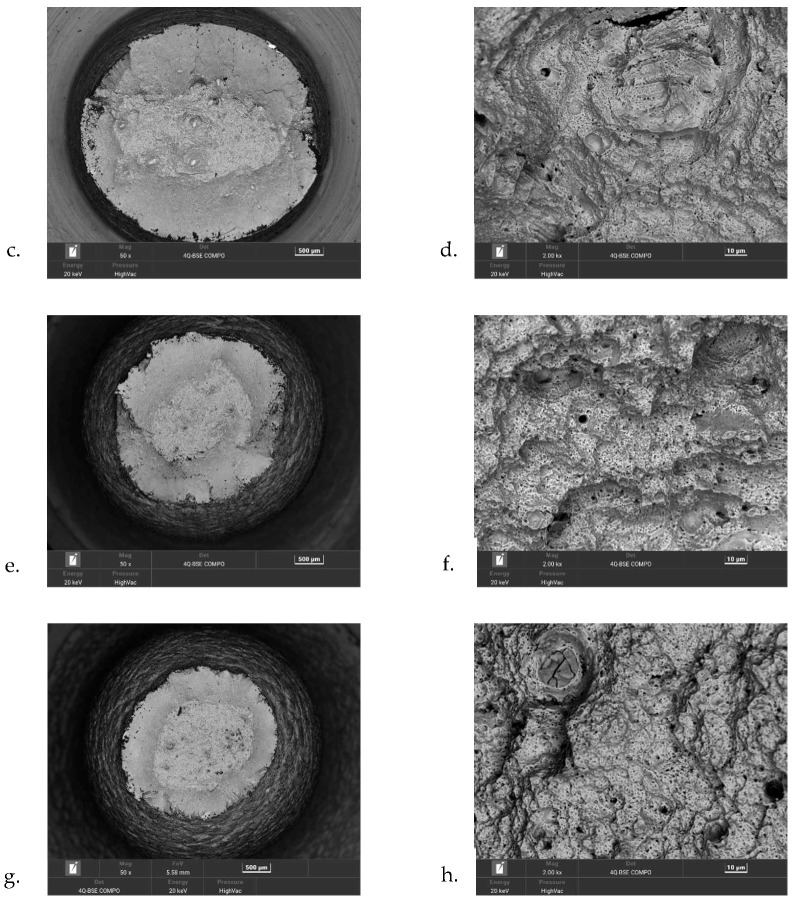
Images of the fracture surface morphology of samples taken with a scanning microscope after static tensile testing was conducted: (**a**,**b**)—200 °C; (**c**,**d**)—300°C; (**e**,**f**)—470 °C; (**g**,**h**)—550 °C.

Figure 5 shows images of the fracture surfaces of the samples tested at 200, 300, 470, and 550 °C. The magnification of the images was chosen so that the entire fracture surface morphology of the sample would be visible in the photos (Figure 5a,c,e,g). Figure 5b,d,f,h show a representative cross-sectional view of the fracture using higher image magnification. In Figure 5a,c,e,g, it can be observed that, as the test temperature increased, the cross-sectional area of the specimens at the rupture region decreased. At the test temperatures of 200 and 300 °C, the images showed areas where a brittle fracture occurred (Figure 5b,d). After the testing at 470 and 550 °C, the fracture surface of the samples showed large areas of plastic fracture. The mechanism of plastic fracture involves nucleation, growth, and the coalescence of voids. This type of decohesion occurs in the central part of the fracture surface of the samples clearly visible in Figure 5a,c,e,f. In contrast, the fracture at the periphery of the specimen, formed by shear action, shows strongly it is deformed and is not preceded by nucleation of voids on inclusions. Figure 5b,d,f,h show an increase in the area where the dimples occur as the test temperature increases. A significant change in the type of fracture occurs at a test temperature of about 470 °C. At this temperature, the beginning of a decrease in the strength of the steel is also observed.

### 4.3. The Results of Numerical Calculations

#### 4.3.1. Temperature Field in Core

**Figure 6:**
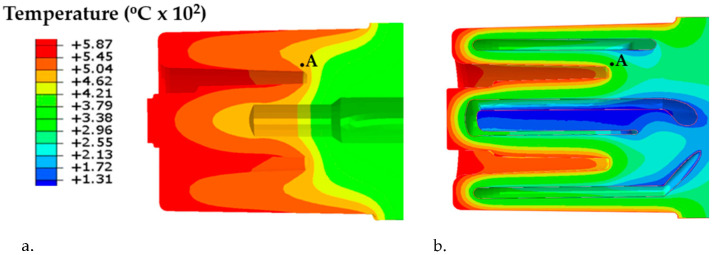
A comparison of temperature field in the cores: (**a**) classical (6.5 s after injection); (**b**) conformal (2.8 s after injection) when the temperature at the point marked ‘A’ reached its highest value during the stabilised core cycle.

Determining the fatigue life of the core with the conformal cooling system first required determining the temperature changes in the core and the instantaneous values of the stress-state components using the FEM method. The calculations were carried out using Abaqus 2019 based on the model described in Section 3.1. As mentioned in the introduction, the design of the conformal cooling channels differed from the traditional solution in having smaller channel diameters and a much smaller distance between the channel axis and the mould cavity surface. This had a strong influence on the temperature variations inside the core during the successive phases of the pressure mould cycle. A comparison of the temperature field in the cross-section of two cores with the same external shape, but with two different cooling methods is shown in Figure 6. Figure 6a shows the temperature field in the axial cross-section of the core with the conventional linear channel obtained by drilling. Figure 6b, on the other hand, shows the map of the temperature field in the same longitudinal cross-section of a core made by SLM with a conformal cooling channel. This allows for an easy comparison of the instantaneous temperature values in the presented cross-sections of both cores when the temperature at the point marked “A” (located at a distance of 0.5 mm from the core/alloy contact surface) reached its highest value during the core cycle. The temperature values were much lower in the cross-section of the core with the conformal cooling channel (Figure 6b) as compared to the corresponding positions of the core zones cooled by the traditional method (Figure 6a).

**Figure 7:**
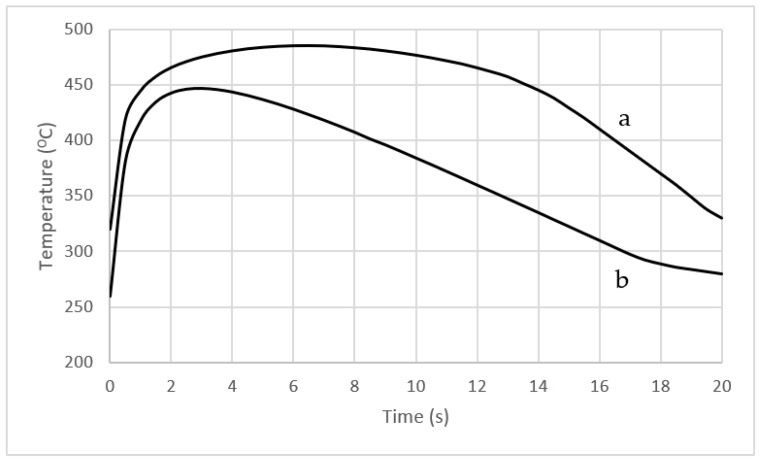
Temperature variation as a function of time numerically determined for Point “A” (Figure 6) in cores: (a) with a conventional cooling channel; (b) with a conformal cooling channel.

A comparison of the temperature changes in the two cores from the moment the mould was filled to the moment it was opened at the point marked ‘A’ (Figure 6) is shown in Figure 7.

The course of these changes is representative of most areas inside and on the surface of cores with conformal and traditional cooling channels. When a conformal cooling system is used, the core temperature is lower during the HPDC process cycle as compared to the operating temperature of a core that is cooled using a traditional linear channel (Figure 7). In addition, a conformal cooling system results in a much faster temperature decline at the point of operation as compared to a traditional system (Figure 7, curve b). In contrast, when a traditional cooling channel is used, the temperature decreases much more slowly (Figure 7, curve a).

#### 4.3.2. The State of Stress in Core

**Figure 8:**
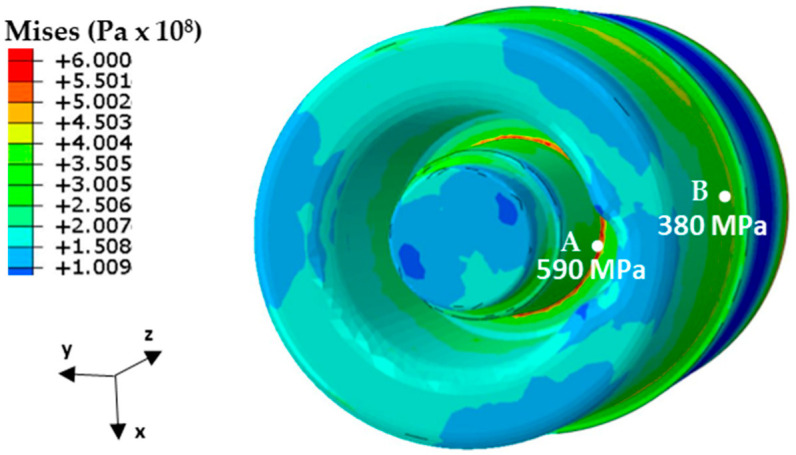
The Mises stress on the core surface at 2.8 s after metal injection into the mould.

Temperature changes during HPDC process cycles are responsible for the time-varying state of the strain and stress in the core. These changes concern both the values and the strain and stress areas. The stress-state components were determined at intervals of 0.5 s. on the basis of the model described in Section 3.2. Figure 8 shows the reduced Mises stress values occurring on the core surface 2.8 s after the metal was injected into the pressure mould. After this time, the stresses reached their highest value during the entire mould cycle. The highest numerically calculated value of the reduced stress was 590 MPa at the point located in the rounded area of the two core walls inclined toward each other at an angle of 95° (marked ‘A’) and 380 MPa on the core surface at Point ‘B’, (Figure 8).

**Figure 9:**
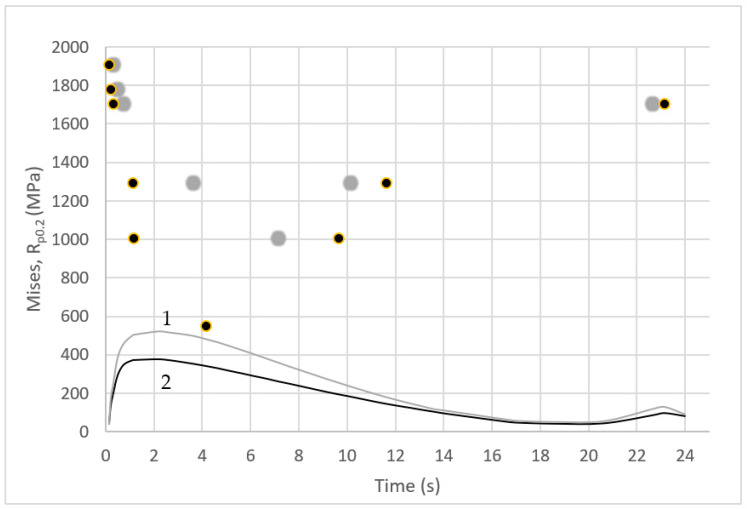
Changes in the Mises stress values occurring on the core surface during one cycle of the pressure machine: 1—near the notch (Point A of Figure 8); 2—on the external surface of the core (Point B of Figure 8) related to the instantaneous yield strength R_p0.2_ (grey and black points corresponding to colours of curves 1 and 2) determined on the basis of the numerically determined core temperature and the results of tensile test within the temperature range of 20–550 °C.

Figure 9 shows the changes in the Mises stress at the core surface at Points “A” and “B” during the three consecutive phases of the mould cycle: crystallisation and cooling of the melt, mould opening, and spraying. The same figure also shows the instantaneous yield stress values R_p0.2_ of the steel at Points “A” and “B”. The values of R_p0.2_ in Figure 9 depend on the core temperature, which varied during the work cycle. The yield strength values of R_p0.2_ were taken from the experimental tests (Figure 4).

In contrast, that point in the cycle at which the core temperature at Points ‘A’ and ‘B’ was the same as the temperature at which the tensile tests were carried out was determined from the numerical calculations. From Figure 9, it is possible to read the difference between the instantaneous value of the Mises stress in the core (Points “A” and “B”) and the experimentally determined yield stress R_p0.2_, which changed in the core during the cycle (Points “A” and “B”) as the temperature changed. During the mould cycle, the instantaneous Mises stress value at Points “A” and “B” of the core did not exceed R_p0.2_ despite the yield stress value decreasing with increasing temperatures.

**Figure 10:**
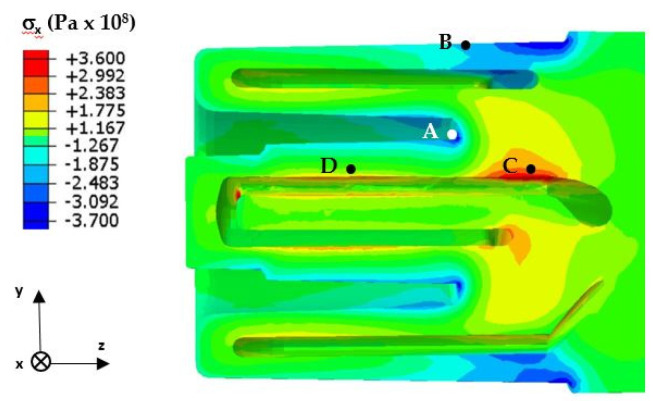
The stress σ_x_ in the y–z section of the core at 0.7 s after metal injection into the mould.

The determination of the Mises stress variation was not sufficient to predict the strength for the fatigue phenomena that occurred in the pressure mould. Therefore, the numerically determined components of the stress state in the core during the pressure-mould-operating cycle were analysed. Given that the fatigue life of the core is determined by the stresses occurring at the core surface and at the surface of the cooling channels, particular attention was paid to the changes in the stress state components in these areas. The near-surface area was dominated by a biaxial stress state defined by the radial x-component and the axial z-component of the stress. The values of the x-component at 0.7 s after metal injection are shown in Figure 10. In Figure 10, the absolute values of the stress (colour-coded red and blue) were highest at the core and channel surfaces. In the area near the core surface, there were compressive stresses, while in the zone close to the surface of the cooling channels, there were tensile stresses.

**Figure 11:**
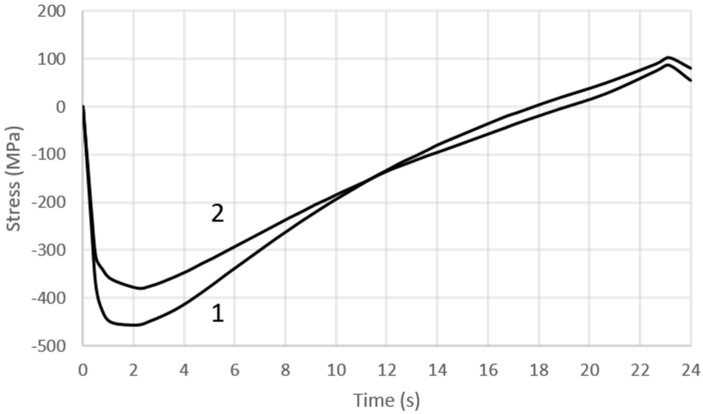
The variation of the stress state component σ_x_ on the outer surface of the core: 1—in the vicinity of the notch (Point A of Figure 10); 2—on the surface (Point B of Figure 10).

**Figure 12:**
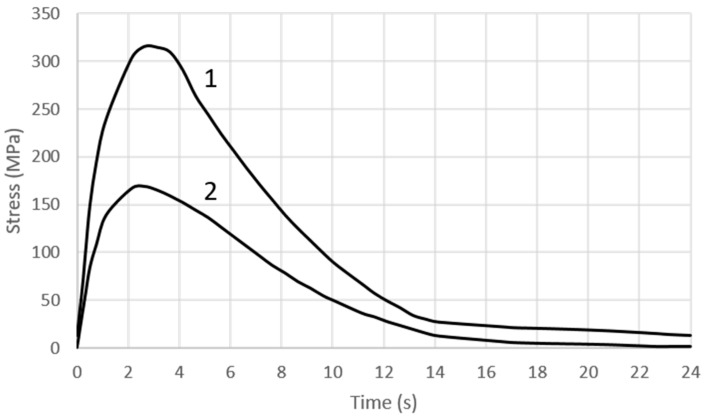
Changes in the stress-state component σ_x_: 1—in the area of change of the cooling channel trajectory; 2—on the surface of the rectilinear section of the cooling channel.

A detailed analysis of the stress variation was possible on the basis of the diagram of its variation during the mould cycle (Figure 11 and Figure 12). Figure 11 shows the changes in the stress state component x in the vicinity of the core/metal alloy contact surface—both where the notch was present (Figure 10, Point A) and where the shape of the core did not cause a local stress pile-up (Figure 10, Point B). In both cases, the changes in the stress determined as a function of time were similar except for the higher stress values that were found in the vicinity of the notch (Figure 11). After the metal was injected into the mould, there was a rapid increase in the compressive stress; this lasted for about 2.5 s (after which, the stress values decreased and changed from compressive to tensile when the mould was opened). The maximum tensile stress values occurred at the moment of spraying with the cooling liquid.

Figure 12 shows the variation of the stress state component σ_x_ in the vicinity of the surface of the cooling channel—both where a change in its trajectory occurred (Figure 10, Point C) and where the channel was rectilinear (Figure 10, Point D). In the vicinity of the cooling channel, there were tensile stresses—the value of which increased and reached a maximum at about 3.5 s after the metal was injected into the mould. After this time, the stresses decreased in value, and the opening of the mould and spraying had little effect on their changes (Figure 12).

The analysis of the stress changes presented above for selected areas of the core (Points A, B, C, and D) was considered to be representative for the other areas. Naturally, differences will occur if we carefully analyse the stress values in different areas of a core.

#### 4.3.3. The Fatigue Life of the Core

To carry out a fatigue life analysis based on fe-safe, it is necessary to define the material parameters and select a fatigue criterion. A detailed description of the material parameters needed to formulate the problem and the algorithm used for the fatigue life prediction is presented in Section 3.3. The algorithm used in the calculations (based on the concept of a critical plane and the hypothesis of maximum principal strain) is usually applied to brittle materials. Carrying out the calculations requires the definition of the fatigue cycle. It was assumed that the fatigue cycle is defined by two stress states occurring at the core surface: the stress state 2.5 s after the metal is injected into the mould, and the stress state at the moment of spraying the core surface with liquid. At these two times, the compressive stresses and the tensile stresses at the contact surface of the core with the metal alloy reach a maximum value (Figure 11). The fatigue cycle for the channel surfaces is, in turn, defined by the stress state occurring 3.5 s after the metal is injected into the mould and the stress state immediately before the mould closes (Figure 12). This is when the changes in the tensile stress values have the greatest amplitude. The number of cycles predicted for crack initiation is obtained as a result of the calculations characterising the fatigue life of the core.

**Figure 13:**
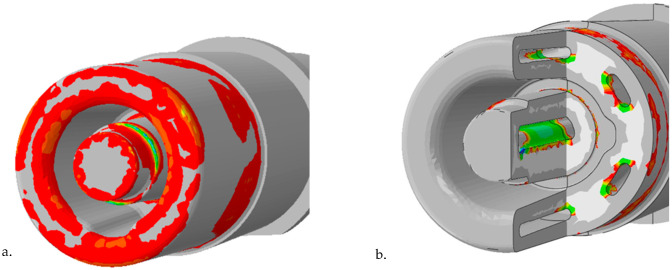
Colour-coded numerically defined zones with the lowest fatigue life: (**a**) outer surfaces of the core (110,000 to 150,000 cycles); (**b**) the cooling channel surfaces (80,000 to 120,000 cycles).

Fatigue life calculations were carried out twice, assuming a roughness of Ra = 0.4 μm and Ra = 10 μm for the model surface for the two calculations. In this way, the difference between the roughness of the outer surfaces of the machined core and the channel whose surface retained the roughness resulting from the SLM process was considered. Figure 13a shows the zones of the outer surfaces of the core with limited fatigue life. Those areas with a fatigue life of 150,000 cycles are coloured red, while those with a predicted fatigue life of 110,000 are coloured blue. In contrast, Figure 13b shows the areas of limited fatigue life occurring on the channel surface. The red colour indicates those areas with a life of 120,000 cycles, while the green colour indicates those areas with a life of 80,000 cycles of the pressure mould.

## 5. Discussion of Results

Experimental tests and numerical simulations were carried out to determine the stress field and fatigue life of an SLM-made core of maraging 1.2709 steel powder forming part of a HPDC mould. In order to increase the efficiency of the heat transfer from the solidifying alloy and reduce the percentage of the porosity in the casting, a drilled linear cooling channel was replaced by a conformal channel. With its trajectory and shape, this channel was matched to the shape of the external surfaces of the core. In order to compare the performance of the traditional and conformal cooling systems, Figure 6 shows the temperature fields in the axial-sections of both cores when the temperature reached its highest values during the pressure machine cycle. A large temperature difference between the two cores can be seen in Figure 6. On the one hand, the use of the conformal channel (Figure 6b) cooled the entire volume of the core by about 70 °C, while on the other hand, its aim was to reduce the temperature locally. The local cooling of the core had a direct effect on the solidification process, causing a reduction in the grain size and the elimination of any porosity. The trajectory of the conformal channel close to Point ‘A’ located in the contact zone between the core surface and the metal (Figure 6b) significantly reduced the core temperature at this point as compared to the impact of the linear channel (Figure 6a). The exact course of the temperature variation at Point “A” is shown in Figure 7. The use of the conformal channel reduced the core temperature at Point “A” during the entire pressure machine cycle as compared to the temperature of the same point in the core cooled in the traditional manner. In addition, the conformal channel resulted in a much faster temperature drop at this point when compared to the traditional one by matching its position and shape to the external surfaces of the core (Figure 7b). In contrast, the use of a linear channel extended the duration of the high temperature in this area of the core (Figure 7a).

A detailed analysis of the effect of the operation of the conformal cooling system on the increase in the strength of the casting and the elimination of the porosity was the subject of the authors’ earlier work mentioned in the introduction [1]. This research mainly addressed the issue of thermal stresses in a core with a conformal channel and the prediction of its fatigue life. During the design of the conformal core cooling system, much smaller channel diameters, smaller distances between the axes of the adjacent channels, and much smaller distances of their surfaces from the surface of the mould cavity were used than in the case of traditional linear channels drilled in moulds. Therefore, an increase in the temperature gradient between the mould surface and the channel surface is to be expected because of the significant reduction in the wall thickness. This may have an impact on the increase in the stress values and, consequently, on the fatigue life of the core.

In order to define the mechanical properties of the SLM-produced 1.2709 steel needed to develop a numerical model and calculate the fatigue coefficients using the Seeger approximation method, strength tests were carried out over a range of mould-operating temperatures.

It was important to determine the temperature above which there is a large decrease in the properties of the SLM-produced 1.2709 steel. The tests carried out showed that the tensile strength of the steel decreased by approximately 300 MPa within a test temperature range of 25 to 470 °C. At 470 °C, however, the tensile strength still remained high (UTS = 1600 MPa). Above this temperature, a rapid decrease in strength could be observed. Steel 1.2709 is characterised by a high yield strength, Rp0.2, which is close to the ultimate tensile strength (UTS) up to approximately 470 °C: at 25 °C, Rp0.2 = 0.96 UTS; at 300 °C—Rp0.2 = 0.9 UTS; and at 470 °C—Rp0.2 = 0.82 UTS (which corresponded to approximately 1300 MPa). The results of the strength tests should be related to the operating temperature of the core. The results of the temperature field simulation showed that, in the bulk of the core, the temperature was between 280 and 330 °C.

In contrast, the temperature was above 450 °C in the layers close to the metal contact surface (and even slightly above 560 °C at the contact surface itself). An analysis of the stress and temperature changes in the core during the pressure moulding cycle and a comparison of their values with the yield stress Rp0.2 of the steel at a given temperature showed that the values of the Mises stress on the core surface did not exceed yield stress Rp0.2. Only in the fourth second after metal injection, the Mises stress value at the core surface was close to the yield stress Rp0.2. The difference then was about 80 MPa (Figure 9). Scanning microscope observations showed that, as the temperature at which the tensile test was performed increased, the narrowing of the specimen increased, and the proportion of the brittle fracture visible in images taken at a higher magnification decreased (Figure 5). Contact between liquid metal and a core surface at a much lower temperature than the metal induces a compressive stress field in the immediate vicinity of the heated surface. The reason for this is that the expansion of the surface layers is inhibited by the neighbouring lower-temperature layers at some distance from them. As long as the mould is closed, the decrease in the core surface temperature occurs by the conduction of heat to zones that have lower temperatures. When the mould is opened, this process continues and is further intensified by heat exchange with the surroundings. Spraying a coating to protect the casting from sticking to the surface of the core causes a significant reduction in the temperature of the surface layer. This phenomenon is the cause of tensile stresses on the core surface, which are a reaction to the interaction of the inner layers of the core with the higher temperature at that moment. As a result of the successive heating and cooling phases of the core surface, the near-surface layers are subjected to alternating compressive and tensile forces.

The result of stresses of varying signs is the phenomenon of fatigue, which leads to the initiation and propagation of near-surface cracks. Thermal cracks occur on flat surfaces and do not depend on the shape of the mould, but only on the course of the temperature changes and the properties of the material from which it was made. Stress cracks appear at stress concentrations such as notches, undercut holes, and edges. They are the joint result of the influence of temperature cycling and the accompanying core-shape changes.

Numerical calculations confirmed the occurrence of much higher temperature gradient-induced stresses in the vicinity of the structural notches as compared to the core surface (Figure 9, Figure 10, Figure 11 and Figure 12). Crack initiation took place at the core and channel surfaces. There was a biaxial stress state. From its components, the deformations (including the principal strains used in “maximum principal strain analysis”) were determined. Following the calculations according to the fatigue model described in Section 3.3, the fatigue life-limited areas on the channel core surface were determined. In fatigue life-limited zones occurring at the core/metal alloy contact surface, crack initiation is predicted after approximately 110,000 mould cycles. However, it must be considered that the calculation model does not take into account the effect of the erosion of the surface caused by the physico–chemical interaction of the liquid alloy jet. In reality, this value may be lower. In contrast, crack initiation may occur after approximately 80,000 in the fatigue-limited zones that occur on the surfaces of the cooling channels. The lower number of cycles required for crack initiation on the surface of the conformal channel is due to the frequent change in its trajectory, which causes a local increase in the stress.

The conducted analysis of the fatigue life of the core has its limitations; these are related to the simplification of the three-dimensional geometrical model of the casting and the mould. In the temperature model, the effect of filling the mould with alloy was neglected, assuming that it already had little influence on the result of the stress calculations in the core once the core temperature stabilised after a few cycles of mould operation. It is also difficult to precisely determine the conditions of mechanical contact between the core surface, the mould, and the casting. The material coefficients were adopted using ProCast databases and our own research. In the fatigue model, the fatigue coefficients therein were determined on the basis of a static tensile test carried out within the mould-operating-temperature range using the Seeger approximation. As a continuation of the research, it is planned to compare the fatigue life determined from the numerical model of the core with the actual course of fatigue phenomena occurring during the casting and to supplement (or correct) the current model on the basis of the acquired information.

## 6. Conclusions

This paper presents an analysis of the fatigue life of a pressure mould core carried out using the Abaqus and fe-save programmes. The three-dimensional numerical model largely takes the geometry, material properties, and technological process of the working pressure mould core made by the SLM method with a conformal cooling channel into account. The calculations were based on a thermal, stress, and fatigue model. In the fatigue model, the results of a static tensile test carried out on SLM-made specimens of 1.2709 steel powder within the working temperature range of the pressure mould were used to determine the fatigue parameters using the Seeger approximation method.

By analysing images of the fractures of the specimens using a scanning microscope (which showed features of both brittle and plastic fracture), a biaxial fatigue criterion based on the maximum principal strain hypothesis was adopted for the fatigue calculations. The analysis of the components of the stress state in the core during the pressure mould cycle indicated the presence of both compressive and tensile stresses on the core surface. The compressive stresses dominated for almost the entire duration of the process except when the mould was opened and its surface was sprayed with release fluid. Tensile stresses then occurred at the surface. When analysing the core fatigue process, the changes in the Mises stress values can only be regarded as indicative and used to determine the positions of the local stress-increase areas. The Mises stress level peaked at about 400 MPa at the core surfaces during the cycle and increased to about 600 MPa in the vicinity of the notches. However, at no point during the duty cycle did it exceed the yield stress value.

The surfaces of the cooling channel were subjected to tensile stresses throughout the pressure machine cycle. The core surfaces were subjected to asymmetric cycles of alternating compression and tension, while the channel surfaces were subjected to an alternating tensile cycle. On the channel surfaces where the trajectory changes caused a local build-up of tensile stresses (to a value of about 350 MPa), crack initiation was predicted after about 80,000 cycles. On the other hand, the durability was 110,000 cycles on the surface. However, it must be realised that the calculation model did not take the effect of the erosion of the mould surface caused by the physico–chemical interaction of the metal jet into account. In reality, this value may be lower. Subsequent work will concern verifying the correctness of the assumptions that were made in the calculation model based on metallographic and mechanical tests of the material of cores after being removed from the mould after several tens of thousands of cycles.

## Figures and Tables

**Table 1:** The chemical composition of the studied powder based on spectral analysis (wt.%).

Ni	Mo	Co	Ti	Cr	Fe
18.14	4.51	8.99	0.71	0.28	balance

**Table 2:** HPDC cycle stages adopted in the thermal model.

Step	Name	Starting Point (s)	Time of Completion (s)
1	Alloy crystallisation and casting cooling	0	22
2	Mould opening and casting extraction	22	23
3	Spraying water suspension	25	27
4	Preparing mould for reclosure	27	35

**Table 3:** Values of heat transfer coefficients h_int_ and h_Surf_ used in the temperature model.

Step	Interaction Pair	*h*_int_ (W/m^2^_*_C) ^1^	*h*_Surf_ (W/m^2^_*_°C) ^1^	T_∞_ (°C)
1, 2	Mould—Cast	50,000 (liquid alloy) 1000 (solid alloy)		
1–4	Channel—Oil		28,500	20
2	Mould—Calm Air		20	100
3	Mould—Lubricant		1000	25

^1^ ProCAST software database.

**Table 4:** Thermophysical properties describing the constitutive model of the cast alloy AlSi9Cu3 and SLM 1.2709 maraging steel mould.

Material	Conductivity		Density		Specific Heat		Latent Heat	
AlSi9Cu3 ^1^	T (°C) λ (W/m_*_K)		T (°C) ρ (kg/m^3^)		T (°C) c (J/kg_*_K)		T (°C) L (kJ/kg)	
	35	136	35	2750	35	869	570	470
	451	155	488	2654	677	1128	580	470
	522	151	550	2610	739	1140		
	600	80	587	2515				
	703	84	700	2475				
X3NiCoMoTi18-9-5 ^2^	20		8025		450			

^1^ ProCAST software database; ^2^ EOS ToolSteel 1.2709 material data sheet.

**Table 5:** Mechanical properties describing the constitutive model of SLM 1.2709 maraging steel.

Temperature	25 °C	200 °C	300 °C	470 °C	500 °C	550 °C
UTS (MPa) ^1^	1988	1959	1886	1577	1444	1040
R_p0.2_ (MPa) ^1^	1906	1780	1703	1294	1003	560
Young’s modulus (MPa) ^1^	190,000	190,000	190,000	180,000	162,000	155,000
Poisson’s ratio ^2^	0.33	0.33	0.33	0.33	0.33	0.33
Temperature Range	25–100 °C	25–200 °C	25–300 °C	25–400 °C	25–600 °C	
Linear expansion coefficient (1/K) ^2^	10.72 × 10^−6^	10.15 × 10^−6^	11.50 × 10^−6^	11.51 × 10^−6^	11.55 × 10^−6^	

^1^ Experimental research; ^2^ EOS ToolSteel 1.2709 material data sheet.

**Table 6:** Mechanical properties describing fatigue model SLM 1.2709 maraging steel.

Temperature	25 °C	200 °C	300 °C	470 °C	500 °C	550 °C
Fatigue-strength exponent *b*	−0.087	−0.087	−0.087	−0.087	−0.087	−0.087
Fatigue-strain exponent *c*	−0.58	−0.58	−0.58	−0.58	−0.58	−0.58
Fatigue-strength coefficient σ*_f_*^′^	2982	2938.5	2829	2365.5	2166	1560
Fatigue-ductility coefficient ε*_f_*^′^	0.039592	0.050849	0.079184	0.165118	0.153873	0.316411
Strain-hardening coefficient *K′* (MPa)	3280.2	3232.35	3111.9	2602.05	2382.6	1716

## Data Availability

The data that support the findings of this study are available from the corresponding authors, [J.P.; A.B.; A.G.-K.], upon reasonable request.
